# Bifunctional hairy silica nanoparticles as high-performance additives for lubricant

**DOI:** 10.1038/srep22696

**Published:** 2016-03-03

**Authors:** Tianyi Sui, Baoyu Song, Yu-ho Wen, Feng Zhang

**Affiliations:** 1 Harbin Institute of Technology, School of Mechatronics Engineering, Harbin, 150001, China; 2 National Synchrotron Radiation Research Center, Experimental Facility Division, Hsinchu, 30076, Taiwan

## Abstract

Bifunctional hairy silica nanoparticles (BHSNs), which are silica nanoparticles covered with alkyl and amino organic chains, were prepared as high-performance additives for lubricants. Compared with hairy silica nanoparticles covered by a single type of organic chain, binary hairy silica nanoparticles exhibit the advantages of both types of organic chains, which exhibit excellent compatibility with lubricants and adsorbability to metal surfaces. Nanoparticles with different ratios of amino and alkyl ligands were investigated. In comparison to an untreated lubricant, BHSNs reduce the friction coefficient and wear scar diameter by 40% and 60%, respectively. The wear mechanism of BHSNs was investigated, and the protective and filling effect of the nanoparticles improved because of collaboration of amino and alkyl ligands.

During the past two decades, hairy nanoparticles (HNPs) have been broadly investigated and applied in a variety of fields because of their unique properties^1^,^2^,^3^,^4^,^5^,^6^,^7^. In particular, hairy silica nanoparticles (HSNs) are important because of their economic efficiency and eco-friendliness. Silica nanoparticles have been investigated on their tribology properties as additives in lubricants, and to facilitate satisfactory nanoparticle dispersion, nanoparticles have been modified using various surface modifying agents^8^,^9^,^10^,^11^,^12^,^13^,^14^,^15^,^16^,^17^. Among these nanoparticles, amino-functionalized HSNs exhibit excellent anti-wear and friction reduction properties because of their good adsorbability on metal surfaces^18^,^19^. However, when these amino-functionalized nanoparticles were added to lubricants, poor dispersity and concentration-sensitive performance were observed because of aggregation of the amino groups and the incompatibility of the amino groups with the organic solvent^19^,^20^. Grafting long alkyl chains onto a nanoparticle surface is an effective method for preventing aggregation and enhancing the compatibility of HSNs in organic solvents^21^,^22^,^23^. Therefore, bifunctional HSNs may be able to address the aforementioned limitation of conventional amino-functionalized nanoparticles by grafting both amino and alkyl chains onto silica nanoparticles.

As a promising nanomaterial, bifunctional nanoparticles exhibit special properties because they integrate the advantages of two types of ligands. Bifunctional nanoparticles have exhibited excellent performance when applied in the electrical and biomedical fields^24^,^25^,^26^,^27^,^28^,^29^,^30^,^31^,^32^. Therefore, the tribological performance of bifunctional hairy silica nanoparticles (BHSNs) is important to investigate because notable disadvantages have been reported for the HSNs with a single type of t ligands. Because of cooperation between the long alkyl chains and amino functional groups, the anti-wear and friction reduction properties of nanoparticles could be improved using the good lubricant compatibility of the alkyl ligands and the metal adsorbability of the amino groups. In this study, bifunctional hairy silica nanoparticles were prepared for use as high-performance lubricant additives. BHSNs that were grafted with different ratios of alkyl and amino ligands were prepared and characterized by scanning electron microscopy (SEM), X-ray diffraction (XRD), Fourier transform infrared spectroscopy (FTIR), X-ray photoelectron spectroscopy (XPS), thermogravimetric analysis (TGA), dynamic light scattering (DLS) and zeta-potential analysis. The tribological properties of BHSNs were investigated using a four-ball tribometer, and the tribological performances of the BHSNs with different ratios of alkyl chains and amino groups were investigated. The A2O1-BHSNs exhibited the best concentration stability and tribological properties, which reduced the friction coefficient by 40% and the wear scar diameter by 60%. The wear mechanism of the BHSNs is discussed, and the excellent tribological performance of the BHSNs may be due to the combined effect of the alkyl and amino ligands, which improves the protective and filling effect of the nanoparticles.

## Results

**Figure 1:**
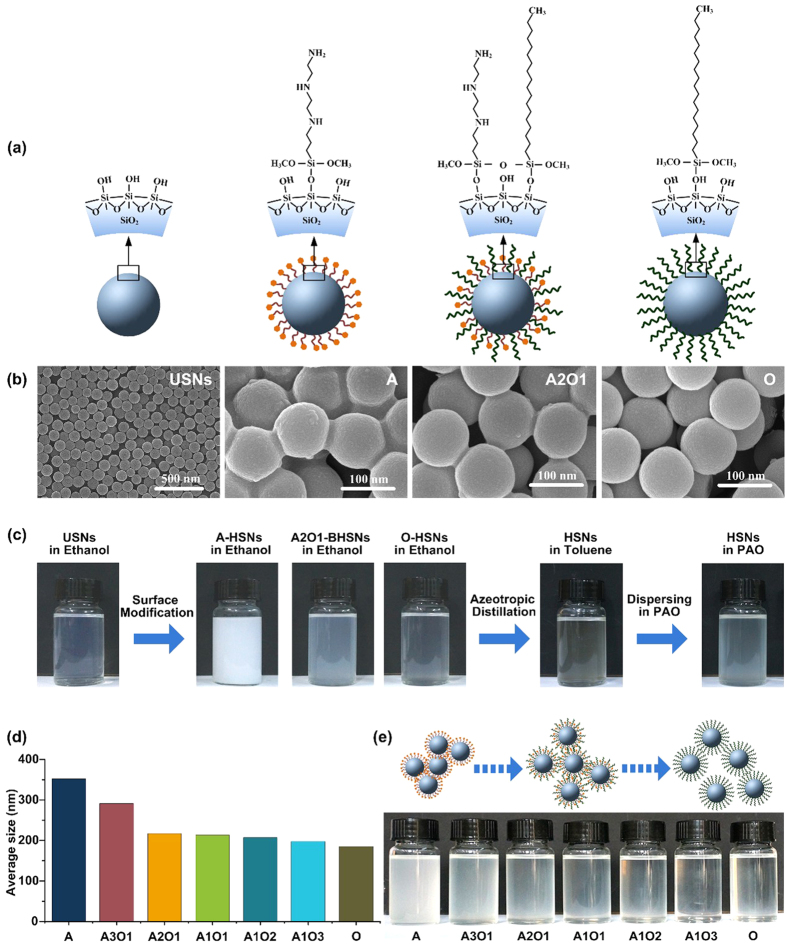
(**a**) Schematic of USNs, A-HSNs, A2O1-HSNs and O-HSNs. (**b**) SEM micrographs of USNs, A-HSNs, A2O1-HSNs and O-HSNs. (**c**) Preparation process of the HSN-PAO lubricant. (**d**) Average particle size of the HSNs in the HSN-PAO lubricants. (**e**) Photographs of different HSN-PAO lubricants. The dispersity of the HSNs was improved by the introduction of O-ligands.

HSNs were synthesized using a simple process. Briefly, unmodified silica nanoparticles were prepared using the Stöber method^33^. Tetraethylorthosilicate (TEOS) was added to a mixture consisting of ethanol, water and aqueous ammonia. Then the solution was stirred at 40 °C for 3 h. These unmodified silica nanoparticles (USNs) were modified using different ratios of *N*^1^-(3-Trimethoxysilylpropyl)diethylenetriamine (DETAS) and octadecyltrimethoxysilane (ODTES). BHSNs with different ratios of DETAS and ODTES chains are referred to as A*m*O*n* BHSNs, where A represents the DETAS, O represents ODTES and *m* and *n* represent the ratio of the two types of silanes. For example, A2O1-BHSNs represent the BHSNs modified with a 2:1 mixture of DETAS and ODTES. Figure 1(a,b) shows schematics and SEM micrographs, respectively, of the USNs and A-, A2O1- and O-HSNs, respectively. The USNs were well separated from each other with excellent spherical morphology, the nanoparticles possessed an average diameter of 100 nm. Different types of HSNs were examined using high-resolution SEM with a higher magnification. The A-HSNs aggregated after surface modification, and strong connections were observed between nanoparticles in the micrograph. As shown in Fig. 1(c), the ethanol solution containing A-HSNs after modification also became cloudy, indicating amino-functional-group-induced aggregation of the A-HSNs. The aggregation was substantially reduced after the introduction of mixed ligands onto the nanoparticles’ surface. O-HSNs exhibited excellent dispersity, with most of the nanoparticles being well separated from each other. Clear image contrast among the three types of HSNs revealed that the connections between the nanoparticles became weak, the aggregation caused by the amino functional groups was alleviated after the introduction of alkyl chains. A photograph of nanoparticles dispersed in ethanol after the modification process (Fig. 1(c)) also indicates that the dispersion of the HSNs was improved after the introduction of the mixed ligands. This improvement in the nanoparticle dispersity was confirmed by the dynamic light scattering (DLS) results shown in Fig. 1(d). Before the tribology test, the BHSNs were dispersed into PAO lubricant using the two-step process shown in Fig. 1(c). Briefly, using azeotropic distillation, BHSNs were transferred from ethanol to toluene and then dispersed into the PAO lubricant by vigorous stirring. The solvent was evaporated by heating in a convection oven. The nanoparticles were dispersed in ethanol, toluene or PAO during the entire preparation process, which is beneficial to their dispersity in the lubricants. The dispersity of BHSNs in PAO lubricants was investigated by dynamic light scattering (DLS). The average size of BHSNs nanoparticles is shown in Fig. 1(d), and the corresponding photograph of BHSN-PAO is shown in Fig. 1(e). As the ratio of ODTES increased, the dispersity of BHSNs in the PAO lubricant significantly improved. The average HSN particle size decreased from 350 nm to approximately 200 nm when the A:O ratio was less than 2:1; the corresponding photograph also shows a substantial improvement in the optical transparency of the lubricant, consistent with the DLS results. The long term stability of the BHSN-PAO lubricants was also investigated by determining the HSN particle size after 4-months of storage of the BHSN-PAO lubricants (Figure S3). The particle size of the A-HSNs increased by 57% after 4 months standing, and the O-HSNs and most of the BHSNs increased by approximately 20% (A3O1 increase by 33%). The introduction of ODTES increased the dispersity and stability of HSNs in PAO lubricant, which was due to the good compatibility of alkyl ligands in lubricants.

**Figure 2:**
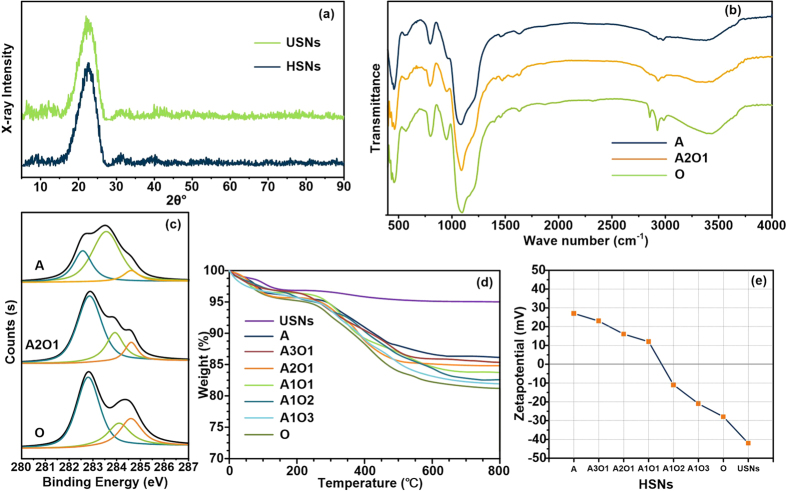
Characterization of BHSNs. (**a**) XPS C 1s spectrum of A, A2O1 and O-BHSNs; (**b**) TGA of different types of BHSNs; (**c**) XRD patterns of USNs and HSNs; (**d**) FTIR spectra of different types of HSNs; and (**e**) zeta potentials of different types of HSNs.

The silica nanoparticles were further characterized by XRD. As shown in Fig. 2(a), the XRD pattern of both the USNs and the HSNs exhibited a broad peak corresponding to silicon. On the basis of the SEM micrographs and XRD patterns, the silica nanoparticles retain their structure and size integrity after modification. Next, the BHSNs were characterized by FTIR spectroscopy; the result are shown in Fig. 2(b). The spectra of all of the different types of HSNs exhibited a strong band at 1010–1100 cm^−1^, which corresponds to the Si-O-Si stretching vibration. The peak at 1620 cm^−1^ for A and 1634 cm^−1^ in the spectrum of A2O1 correspond to N-H bending, and the broad band at 3400 cm^−1^ in the spectrum of A and A2O1 are due to N-H stretching. Peaks located at 2841 cm^−1^ and 2934 cm^−1^ were observed in the spectra of the HSNs and correspond to C-H stretching. In addition, the peak height increased as the ODTES ligand ratio was increased.

The BHSNs were further then characterized by XPS. The survey spectra of the HSNs are shown in Figure S1, the C 1 s subregions of A, A2O1 and O HSNs are shown in Fig. 2(a). The survey spectra show that the N 1 s peak appeared after the introduction of amino functional groups and the C 1 S peak become more intense as the ODTES ratio was increased. The C 1 s subregion of the HSNs was deconvoluted into three Gaussian peaks: a typical C-Si peak at 282.7 eV, a C-H peak at 283.9 eV and a C-C peak at 284.6 eV. The C-C peak increased in intensity as the alkyl long chain ratio was increased.

The organic content of the BHSNs was characterized using TGA. As shown in Fig. 2(d), the organic chains begin to degrade at approximately 300 °C and completely degrade at approximately 600 °C; the inorganic silica nanoparticle core (melting temperature: 1650 °C) remained intact at the end of the test. The organic content of the BHSNs was 10% to 15%. On the basis of the Brunauer-Emmett Teller (BET) test results (ESI Figure S2), the grafting density of the BHSNs was estimated to be 1.1–1.3 ligands/nm^2^.

The zeta-potential results of the HSNs are shown in Fig. 2(e). The zeta potential of the USNs was −42 mV; after modification by DETAS and ODTES, the zeta potential changed to +27 mV and −28 mV, respectively. For BHSNs, as the ODTES ratio increased, the zeta potential gradually decreased from +23 mV to −21 mV. On the basis of this characterization, the bifunctional ligands were successfully introduced onto the silica nanoparticle surface.

The rheological properties of the BHSN-PAO lubricants was tested using a Brookfield rheometer. The viscosity of the BHSN-PAO lubricant as a function of temperature is shown in Figure S4(a), and the change in the neat PAO viscosity was less than 10% compared to that of the BHSN-PAO lubricants. However, the viscosity index (Figure S4(b)) slightly increased after addition of the BHSNs, indicating that the lubricant oil exhibited better thermal stability. The viscosity index (VI) is an arbitrary measure of the change in viscosity as a function of temperature; for a higher VI, the change in the viscosity of an oil with temperature is smaller^34^.

**Figure 3:**
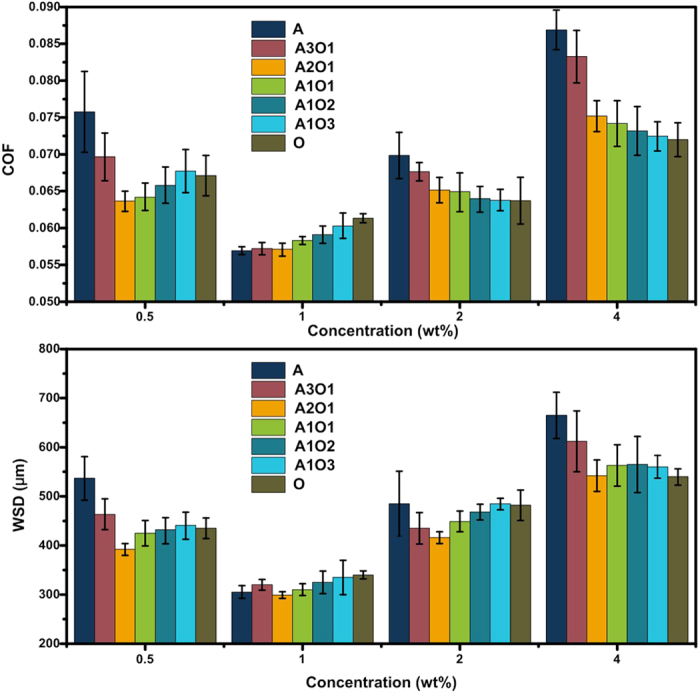
Coefficient of friction (COF) and wear scar diameter (WSD) of different types of BHSNs.

The tribological performance of the BHSN-PAO lubricant was tested using a four-ball tribometer. GCr15 steel bearing balls with a diameter of 12.7 mm were ultrasonically cleaned with ethanol prior to the test. All of the tests were performed at room temperature under a load of 390 N and a rotating speed of 1450 r/min for 30 min, and the coefficient of friction (COF) and wear scar diameter (WSD) were measured. The test results are shown in Fig. 3. The A-BHSNs exhibited excellent anti-wear and friction reduction properties at their optimal concentrations (1 wt%) while the tribological performance was sensitive to the concentration. When more or less nanoparticles were added to the lubricant, the tribological performance deteriorated dramatically. In Comparison to A-HSNs, the O-HSNs exhibited better concentration stability but a higher COF and a greater WSD. When the mixed ligands were introduced onto the nanoparticles’ surface, the tribological performance of the BHSNs exhibited a general trend where the COF and WSD at 1 wt% increased and the concentration stability improved. When the A:O ratio of the HSNs was changed from 1:0 to 2:1, the tribological performance of the HSNs did not at 1 wt% (best concentration) but improves substantially at 0.5, 2 and 4 wt%. However, when the A:O ratio was increased to 3:1, the WSD and COF of the BHSNs began to increase at 1 wt%, and these values remained stable or slightly increase at other concentrations. Among these BHSNs, the A2O1-BHSNs were determined to be the best BHSNs and exhibited excellent tribological performance and concentration stability. In comparison to the untreated pure PAO, which had an average COF of 0.93 and a WSD of 870 μm, the BHSNs reduced the COF by 40% and the WSD by 60%.

## Discussion

**Figure 4:**
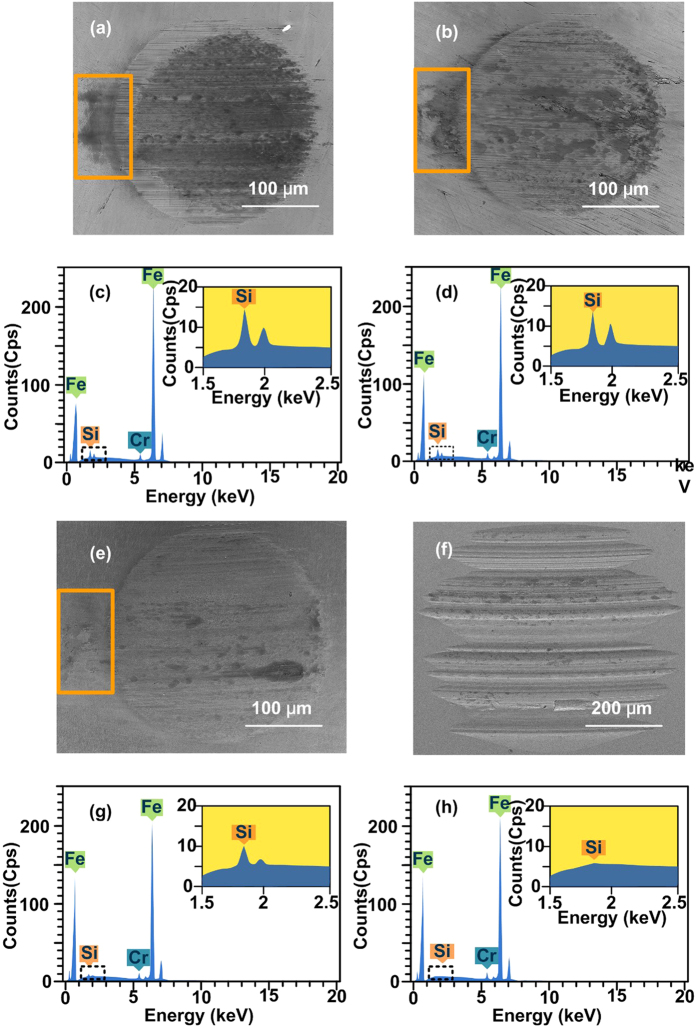
SEM and EDS results for the wear surface of (**a,c**) A-HSNs, (**b,d**) A2O1 HSNs, (**e,g**) O-HSNs and (**f,h**) pure PAO.

**Figure 5:**
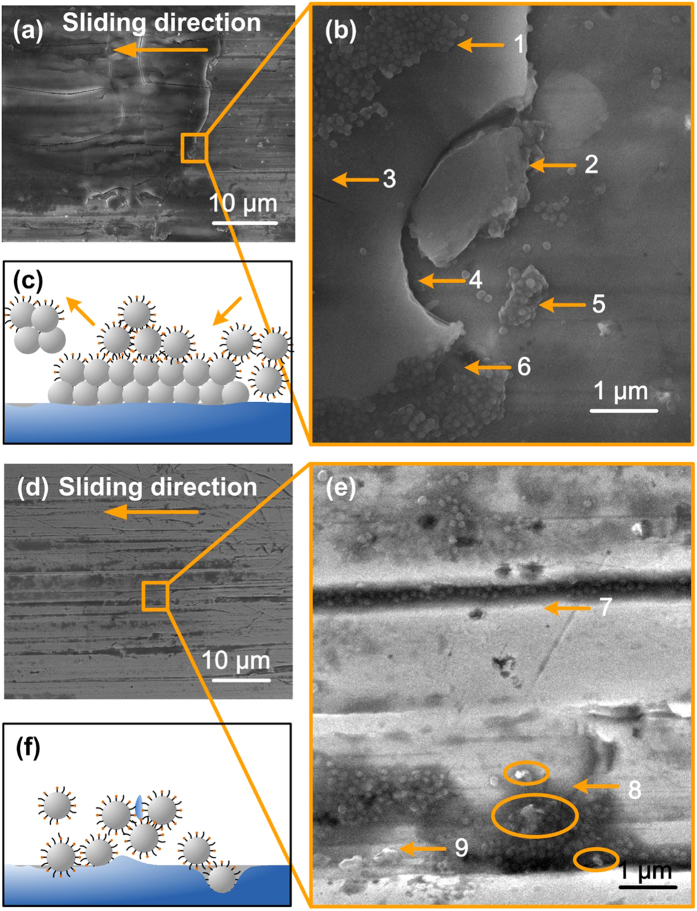
Wear mechanisms of BHSNs: (**a,b**) protective effect of BHSNs; (**c**) a schematic of the formation of the protective effect; (**d,e**) filling and entraining effects of BHSNs; and (**f**) a schematic of the filling and entraining effects.

In this study, we prepared BHSNs with different ratios of amino functional ligands and alkyl ligands and evaluated their tribological performance. On the basis of the results in Fig. 3, both the COF and the WSD of the HSNs decreased when the concentration was increased from 0.5 wt% to 1 wt% and then increased gradually from 1 wt% to 4 wt%. The HSNs at a concentration of 1 wt% exhibited the best anti-wear and friction reduction properties compared to those at lower and higher concentrations because of bad lubrication effects caused by insufficient or excess amounts of nanoparticles in the contact area. The HSNs could not support the surface, form the protective films and efficiently fill in the nano-grooves on the surface when an insufficient amount of nanoparticles were present in the contact area. When the contact area is saturated with nanoparticles, an excess amount of nanoparticles may form large clusters and harm the surface, which is known as three-body abrasion^35^. According to the graph in Fig. 3, larger ratio of DETAS on the nanoparticle surface resulted in better tribological performance of the BHSNs at the optimal concentration (1 wt%). This result is due to amino functional groups that enhance the physical and chemical adsorption of nanoparticles onto the metal surface^36^,^37^. When more nanoparticles are adsorbed, the metal surface is more effectively protected by the BHSNs; consequently, the anti-wear and friction reduction properties of HSNs are improved. This point is further confirmed by the result of the SEM and EDS analyses of the wear surfaces shown in Figs 4 and 5. However, as the DETAS ratio on the nanoparticle surface increased, the tribological performance of the BHSNs became unstable, which resulted in substantial fluctuation of the WSD and the COF with concentration. This behavior was due to the partial aggregation of the nanoparticles resulting from the hydrogen bond between the amino groups^20^. The aggregation may lead to a partially uneven distribution at low concentrations (0.5 wt%) and three-body abrasion at high concentrations (2 wt% and 4 wt%) due to the formation of large clusters of nanoparticles. As shown in the figure, with more O on the nanoparticle surface, the tribological performance of the BHSNs became more stable because of the good compatibility between PAO and the alkyl chains on the HSN surface, which was confirmed by the DLS results. As shown in Fig. 1(d), the average particle size of the HSNs decreased with increasing numbers of alkyl chains on the HSNs’ surfaces.

The optimal A:O ratio was investigated on the basis of the tribology results. The A2O1-BHSNs exhibited the best tribological properties as well as the best concentration stability and lowest COF and WSD. The alkyl ligands significantly improved the tribological performance of the BHSNs in PAO when the A:O ratio was changed from 3:1 to 2:1. The introduction of alkyl ligands substantially increased the concentration stability of the HSNs (the COF and WSD decreased dramatically at 0.5, 2 and 4 wt%). This result may be due to the enhancement of the HSN compatibility in PAO, which improved the dispersity of the HSNs and also maintained the function of the amino groups. Because the surface was still dominated by amino ligands, the amino groups could effectively adsorb and chelate to improve the tribological performance of the HSNs. However, when alkyl ligands dominate the nanoparticle surfaces, the amino functional groups may be covered by long alkyl chains and not function well. Therefore, an increase in the COF and the WSD at all of the studied concentrations was observed when the A:O ratio changed in a stepwise manner from 2:1 to 1:3.

To further characterize the stability of the tribological properties for BHSNs and monofunctional HSNs, we tested the tribological performance of the newly prepared (NP) HSN-PAO lubricant and HSN-PAO lubricant after 1 month of long-term standing (LS). A201-BHSNs, A-HSNs and O-HSNs were compared in the experiment, and the COF and WSD of the HSN-PAO lubricant for both NP and LS are shown in Figure S5. After long-term standing, both the WSD and COF of the HSN-PAO lubricant increased, possibly because of an increase in nanoparticle aggregation. A-HSNs exhibited the largest increase in COF and WSD, and the COF and the WSD of A2O1-HSNs and O-HSNs only increased slightly. On the basis of the test results, the A2O1-BHSNs exhibited better dispersity than A-HSNs but a much lower COF and WSD than the O-HSNs. The A2O1-BHSNs exhibited excellent anti-wear and friction reduction properties as well as excellent stability in their tribological properties upon dispersion in a lubricant.

Figure 4 shows the SEM morphology and EDS spectra of the wear surfaces of A, A2O1, O and pure PAO. These SEM micrographs indicate that the anti-wear property of PAO was dramatically improved after the addition of the HSNs as a lubricant additive. With an increase in the O ratio, the WSD increased and the surface roughness improved. The surface roughness of the wear surfaces was further characterized by laser scanning confocal microscopy (LSCM); the results shown in Table S1 indicate that the surface roughness decreased as the O ratio increased, which may be due to improvement in nanoparticle dispersion. A Si peak was observed in the EDS spectra in addition to the intense peaks for Fe and Cr from the metal surface. As the A ratio increased, the Si peak became stronger, indicating stronger adsorption of the silica nanoparticles onto the metal surfaces. As shown in the SEM figure, an interesting “comet tail” was observed in the outlet region of the wear surface; this region faded away as the A ratio was decreased.

To further investigate the wear mechanism of the BHSNs, we examined the wear surfaces of the A2O1-BHSNs using SEM. Figure 5(a,b) show the protective effect of the BHSNs, where (b) is a high-magnification micrograph of the marked region in (a). As shown in Fig. 5(a), the wear surface was covered with a large and thick protective film. In comparison to the metal surface, which consists of nanogrooves and bumps, the protective film was flat and smooth, which can effectively reduce friction and wear.

When examining the edge of the protective film at high magnification (Fig. 5(b)), we noted several interesting phenomena. A large number of nanoparticles were adsorbed onto the film surface (arrow 1) and edge (arrow 6). Nanocracks were observed on the surface (arrow 3), and nanodebris from the protective film breaking down was observed (arrows 2, 5). An intergranular fracture (arrow 4) was also observed in the micrograph. On the basis of these results, we proposed a schematic representation of the BHSN nanoparticle protective film, which is shown in Fig. 5(c). Because the intergranular fracture and nanodebris consist of spherically shaped nanoparticles, the protective film was formed by BHSNs and these nanoparticles retained their structures in the protective films. In comparison to the clean metal surface where a limited amount of nanoparticles were adsorbed, the BHSNs preferentially adsorbed onto protective-film surfaces and edges, possibly because of the attraction among amino functional groups. Nanodebris from the protective films keep breaking down from the film during the wear process, and new film is simultaneously generated. The attraction among the nanoparticles may be beneficial for the formation and regeneration of protective films because of good nanoparticle dispersion. This behavior was confirmed by examining the wear surface of the A-HSNs and O-HSNs (Figure S6). In the absence of good dispersion, a large cluster of A-HSNs could abrade the metal surface. In addition, in the absence of good adsorption, only a few monodispersed O-HSNs would adsorb onto the wear surface.

Figure 5(d,e) show the filling effect and entraining effect of BHSNs, where (e) shows a high-magnification image of the marked region in (d). Grooves caused by ploughing wear were observed along the sliding direction in Fig. 5(d). The grooves were further examined at high magnification, as shown in Fig. 5(e). As shown in this micrograph, a large number of BHSN nanoparticles were adsorbed onto the metal surface. Importantly, a filling effect was observed on the surface (arrow 7) where the nanogroove was filled with BHSNs, and most of the nanoparticles in the groove were monodispersed. Wear fragments were observed on the surface (arrows 8, 9), and some of these fragments were surrounded by BHSNs. Notably, because the metal surface was ultrasonically cleaned prior to the SEM examination, the nanoparticles in the grooves may have been washed away, leaving only the nanoparticles that were deeply adsorbed into the groove. The movement of the nanoparticles in the groove is shown in Figure S7(a,b). On the basis of the results shown in Fig. 5(e), we proposed a schematic representation of the filling and entraining effect of BHSNs. As shown in Fig. 5(f), the BHSNs were anchored into the nanogrooves because of the good adsorption of the nanoparticles. Here, the nanoparticles in the grooves were simplified as a single nanoparticle. However, in a real lubrication process, large numbers of nanoparticles would fill the grooves and, as previously discussed, the nanoparticles on the upper layer would be washed away by ultrasonic cleaning, leaving only the nanoparticles that are deeply adsorbed in the groove. As shown in Figure S7(c,d), during the lubrication process, the nanoparticles in the groove will maintain dynamic balance, where nanoparticles on the surface of the groove are removed by the lubricant and new nanoparticles are adsorbed. With the aid of alkyl ligands, the dispersity of the BHSNs improved dramatically. Monodispersed BHSNs more easily filled the nanogrooves compared to large clusters of nanoparticles resulting from aggregation. With the aid of the amino functional ligands, the adsorption of the nanoparticles was enhanced and the nanoparticles adsorbed more effectively onto the metal surface. This result is confirmed by the results of the filling effect for the A-HSNs and O-HSNs (Figure S6(c,d)). On the basis of the SEM micrographs, nanoparticles clustered and adsorbed onto the flat surface rather than filling the nanogrooves, even though the A-HSNs exhibited good adsorption onto the metal surfaces. The aggregation limited the filling effect of the A-HSNs. The O-HSNs were monodispersed, and only a few nanoparticles were observed in the grooves because of poor adsorption. The nano-wear debris generated by nanopolishing and nanoploughing was surrounded by BHSNs because of the chelation effect between the amino groups and the metal debris. Therefore, the debris was removed by the lubricants rather than harming the metal surfaces. This result is in good agreement with the results for the outlet region of the contact area (marked region in Fig. 4). The wear debris and BHSNs were removed from the contact area and formed a sediment in the outlet area, where these debris formed a “comet tail” in the outlet area. The amount of sediment in the outlet region of the wear surface decreased with decreasing A ratio on the nanoparticle, and the “comet tail” sediment disappeared under pure PAO lubrication conditions. In comparison to A-HSNs and O-HSNs, BHSNs benefited from the advantages of both the amino and alkyl ligands, which enhanced the protective and filling effects of the HSNs.

In conclusion, we prepared BHSNs with different ratios of amino functional and alkyl ligands. The BHSNs exhibited excellent compatibility and stability when dispersed in PAO lubricant. The optimal ratio of BHSNs was investigated, and A2O1-BHSNs exhibited the best tribological properties, including reducing the COF by 40% and the WSD by 60% compared to those of the untreated pure PAO. The BHSNs exhibited excellent tribological performance and retained this tribological performance after long-term standing. The wear mechanism of the BHSNs was studied through an analysis of the wear surfaces. A BHSN protective film was observed on the wear surface, and the film continuously delaminated and regenerated during the wear process. The BHSNs filled the nanogrooves and removed wear debris. BHSNs benefited from the advantages of both the amino and alkyl ligands, which enhanced the protective and filling effects of the HSNs.

## Methods

### Materials

Ethanol (99% purity, Sinopharm), TEOS (99% purity, Sinopharm), ammonium hydroxide (28% purity, Sinopharm), toluene (99% purity, Sinopharm), DETAS (95% purity, Sigma-Aldrich), trimethoxyoctylsilane (97%, Sinopharm), OTES (90%, Sigma-Aldrich) and trimethoxysilylpropoxypolyethyleneoxide (85% purity, Gelest) were used as received.

### Preparation of BHSNs-PAO lubricant

A schematic of the BHSN-PAO lubricant preparation process is shown in Fig. 1(c). Unmodified silica nanoparticles with a diameter of 100 nm were prepared using the Stöber method. TEOS was added to a mixture consisting of ethanol, water and aqueous ammonia, and the resulting mixture was gently stirred at 300 rpm and 40 °C. For the 100 nm silica nanoparticles, a mixture consisting of ethanol, water and aqueous ammonia was prepared by adding 2 wt% of water and 5 wt% of aqueous ammonia to the ethanol. After the mixture was heated to 40 °C, 5 wt% TEOS was diluted and then added to the mixture. The solution became slightly turbid and blue after 3 h, which indicated the formation of 100 nm USNs. After the reaction was complete, the silica nanoparticles were modified with different ratios of DETAS and ODTES. To achieve dense grafting onto the silica nanoparticles, a large excess of silanes was added to the silica nanoparticle solution. The mixture with different ratios of ligands was divided into two aliquots and added dropwise to the solution with rapid stirring. The second aliquot was added 3 h after the addition of the first aliquot. The solution was maintained at 60 °C for 20 h to ensure completion of the modification. The BHSNs were purified by repeated dialysis using a 10,000 MWCO snakeskin dialysis tube to remove impurities and unlinked silanes. After the modification process was complete, the HSNs were transferred from ethanol to toluene by azeotropic distillation. Toluene was added to the HSN-ethanol solution to form a 3/1 mixture of toluene and ethanol. The solution was maintained at 70 °C for 3 h to remove the ethanol. After the azeotropic distillation process, the HSNs were transferred to toluene. PAO was added to the solution with rapid stirring. The solution was then transferred to a convection oven to remove the toluene from the PAO.

### Characterization

The morphology of the silica nanoparticles was examined using SEM with a Hitachi SU8010, and the silica nanoparticles were treated with sputtered gold before the experiment to enhance the conductivity. The XRD patterns of the silica nanoparticles (5 ≤ °2θ ≤ 90) were recorded using a Panalytical Empyrean X-ray diffractometer equipped with a Cu Kα radiation source; the samples were scanned at 25 °C using a 2θ step size of 0.02 and a scanning speed of 2 °/min. The FTIR analysis of the HSNs was performed on a Nicolet IS10 with a spectral range of 400–4000 cm^−1^. The XPS experiments were performed on a Thermo Fisher Scientific 250Xi X-ray photoelectron spectrometer (Al Kα line of 1486.6 eV and 120 W); the ultra-high-vacuum analysis chamber was maintained at a typical base pressure of 5 × 10^−9^ Torr during sample analysis. The surface area of the unmodified silica nanoparticles was characterized using the BET method, and the nanoparticles were pre-treated under vacuum conditions at 150 °C for 2 h prior to the BET test. TGA was conducted using a TA Instruments Q500; the samples were analyzed at a heating rate of 10 °C /min to 800 °C under flowing nitrogen gas. The zeta-potential analysis of the HSNs dissolved in ethanol as well as DLS analysis of the 1 wt% HSNs dissolved in PAO lubricants were conducted using a Malvern Instruments Zetasizer Nano ZS. The wear surface of a steel ball was examined using a field-emission electron microscope (Zeiss Supra 55 Sapphire). The wear surface was ultrasonically cleaned in ethanol for 5 min prior to SEM and EDS characterization. The tribological properties of the BHSNs were investigated using a four-ball tribometer; the test procedure and schematic setup of the tribometer are shown in Figure S8.

## Additional Information

**How to cite this article**: Sui, T. *et al.* Bifunctional hairy silica nanoparticles as high-performance additives for lubricant. *Sci. Rep.* **6**, 22696; doi: 10.1038/srep22696 (2016).

## Supplementary Material

Supplementary Information
