# Supplementary material for: Bifunctional hairy silica nanoparticles as high-performance additives for lubricant
Source: Sci Rep. 2016 Mar 3;6:22696. doi: 10.1038/srep22696 (PMC4776138; doi:10.1038/srep22696)
Supplement: Supplementary Information [file srep22696-s1.pdf]

Bifunctional hairy silica nanoparticles towards high-performance additives for lubricant

Tianyi Sui, Baoyu Song*, Yu-ho Wen, Feng Zhang

Supplementary information

1. XPS survey spectra of BHSNs

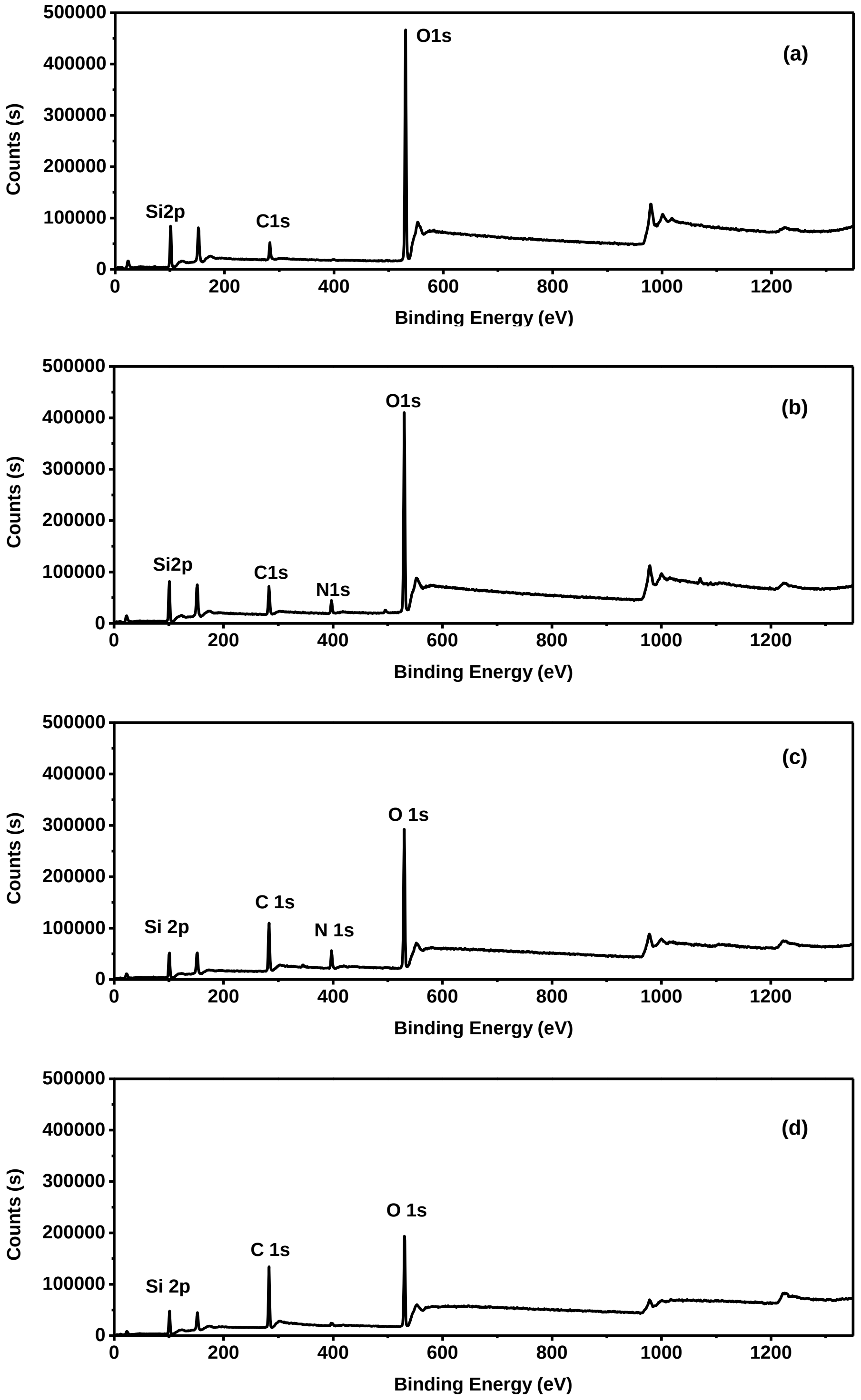

Figure S1. XPS survey spectra of (a) USNs, (b) A-HSNs, (c) A2O1-HSNs, (d) O-HSNs.

2. BET test result for USNs

Slope=12.621,

Correlation coefficient, r=0.999833,

C constant=57.398

Surface Area=271.125 m^2/g

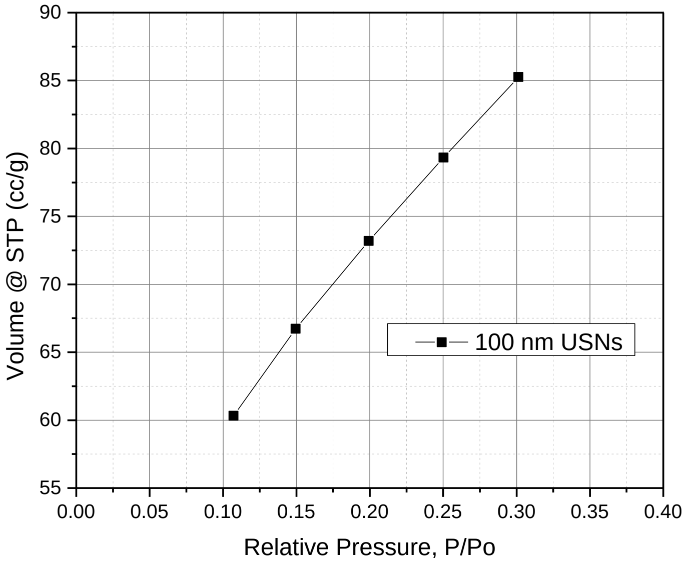

Figure S2. BET test result for 100 nm unmodified silica nanoparticles

3. The stability of HSNs dispersing in PAO lubricants

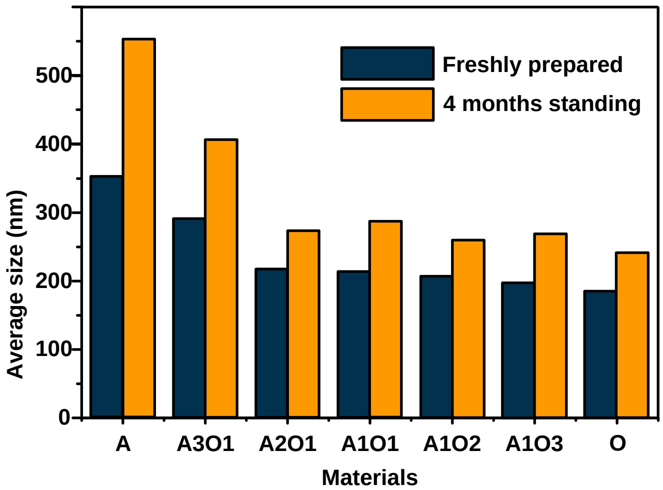

Figure S3. The average size of HSNs dispersing in PAO, the, blue column is the particle size in freshly prepared HSNs-PAO lubricants and the orange column is the particle size after 4 months standing.

4. Viscosity-temperature properties of BHSNs-PAO lubricants

The viscosity index (VI) is an important measure for the change of viscosity with temperature. The higher the VI, the smaller the change of viscosity with temperature. The VI was calculated as following:

$$V = 100\frac{(L-U)}{(L-H)}$$

V indicates the viscosity index, U the oil's kinematic viscosity at 40 °C (104 °F), and L & H are

values based on the oil's kinematic viscosity at 100 °C (212 °F). L and H are the values of viscosity at 40 °C for oils of VI 0 and 100 respectively, having the same viscosity at 100 °C as the oil whose VI we are trying to determine. These L and H values can be found in ASTM D2270. The VI of different types of HSNs was shown in Figure S4(b).

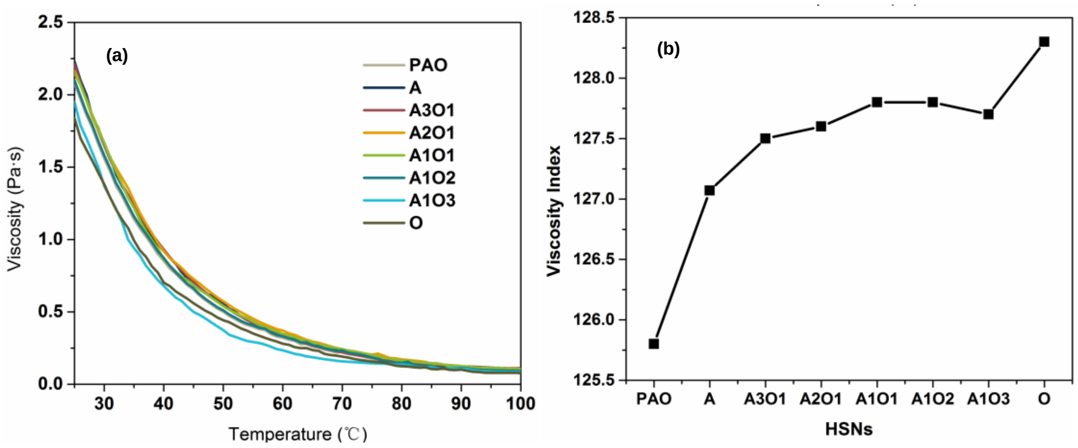

Figure S4. The viscosity-temperature properties: (a) the viscosity versus temperature of different kinds of HSNs-PAO lubricants, (b) the viscosity index of different kinds of HSNs-PAO lubricants

5. The tribological performance stability test of different kinds of HSNs

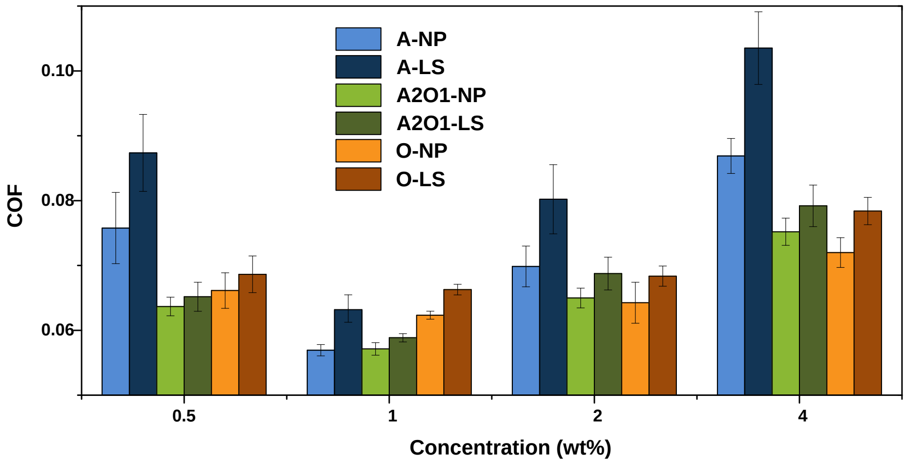

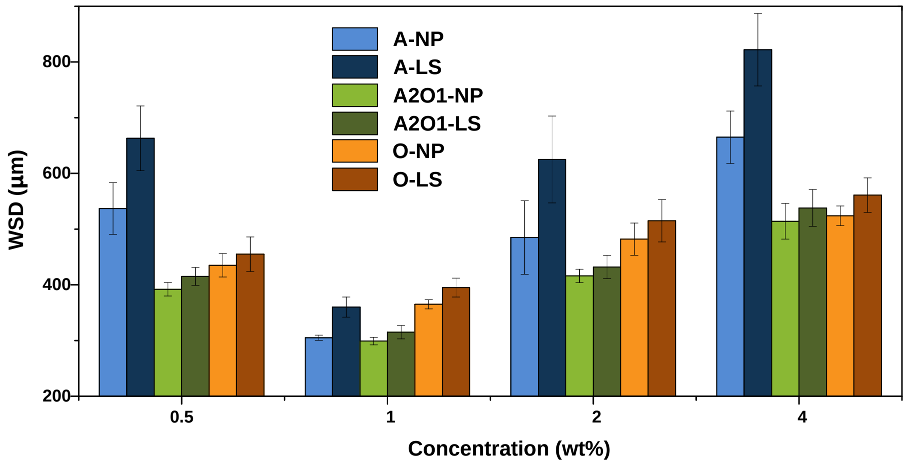

Figure S5. The COF and WSD of A-HSNs, A2O1-BHSNs and O-HSNs for both new prepared HSNs-PAO lubricant and HSNs-PAO lubricant after long term standing

6. Surface roughness of wear surface and surface adsorption

The surface roughness of the wear scar was characterized by laser scanning confocal microscope (LSCM). The surface average roughness (*SRa*) and surface root mean square roughness (*SRq*)of the wear scar surface was shown in table S1. Average roughness is the arithmetic average of the absolute values of the profile height deviations recorded within the evaluation length and measured from the mean line. Root mean square roughness is the root mean square average of the profile height deviations taken within the evaluation length and measured from the mean line (Standard ASME B46.1-2009).

Material	A	A3O1	A2O1	A1O1	A1O2	A1O3	O
SRa (μm)	0.173	0.125	0.073	0.068	0.082	0.075	0.063
SRq (μm)	0.228	0.151	0.091	0.088	0.103	0.092	0.083

Table S1. The surface roughness of different HSNs

7. The wear surface of A-HSNs and O-HSNs

The wear surface of A-HSNs and O-HSNs were examined by SEM and shown in Figure S6 (a, c) and (b, d) respectively. It could be found from the marked region in Figure S6 (a) that large clusters of A-HSNs are found on the wear surface. Grooves are found near the large nanoparticle clusters, which could be due to the ploughing effect of nanoparticles. With amino functional groups on the surface, nanoparticles are easy to aggregate and form large clusters. Those clusters would lead to three body abrasion. FigureS6 (b) shows the wear surfaces of O-HSNs, it could be found that nanoparticles are monodispersed and the wear surface is smoother than the wear surfaces of A-HSNs. However, comparing with A-HSNs which large amount of nanoparticles adsorbed on the surface, only few numbers of O-HSNs are found on wear surface, which could be due to the bad adsorption of alkyl ligands. Nano-grooves were found from Figure S6(c). However, A-HSNs were found to form large cluster nearby instead of filling into the grooves. This could be attributed to the bad dispersion of HSNs that prevent nanoparticles from filling into nano-grooves (the diameter of the grooves is smaller than the diameter of nanoparticle cluster). Although A-HSNs have good adsorption on metal surface, nanoparticles could only adsorbed beside the nano-grooves. With good dispersion, O-HSNs are found mono-distributed on the wear surface, but only few of the nanoparticles are adsorbed on the surface due. With better adsorption and dispersion, BHSNs are found to exhibit better filling effect which monodispersed nanoparticles fill into the grooves and anchored in the grooves.

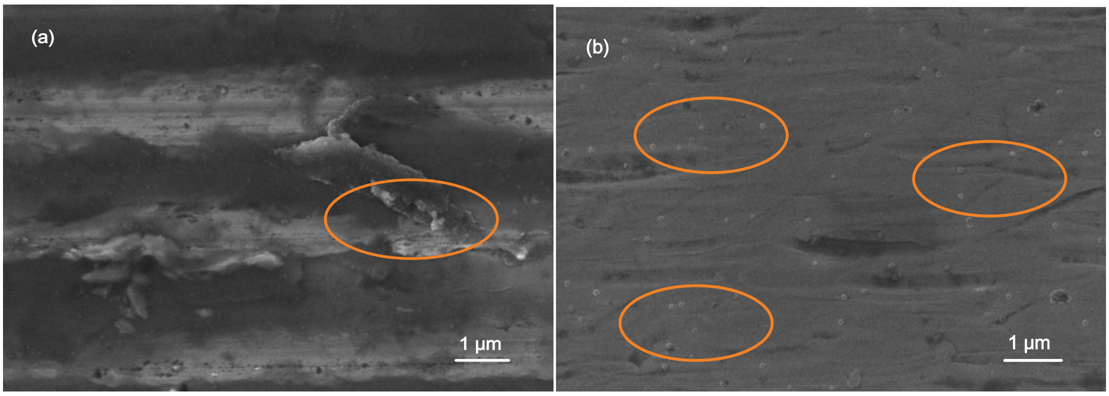

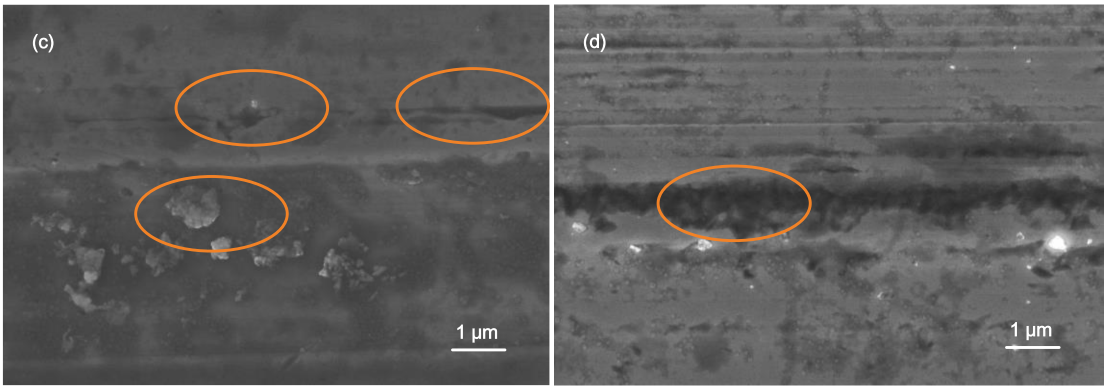

Figure S6. Wear surfaces SEM of for (a, c) A-HSNs, (b, d) O-HSNs

8. The movement of nanoparticles in grooves

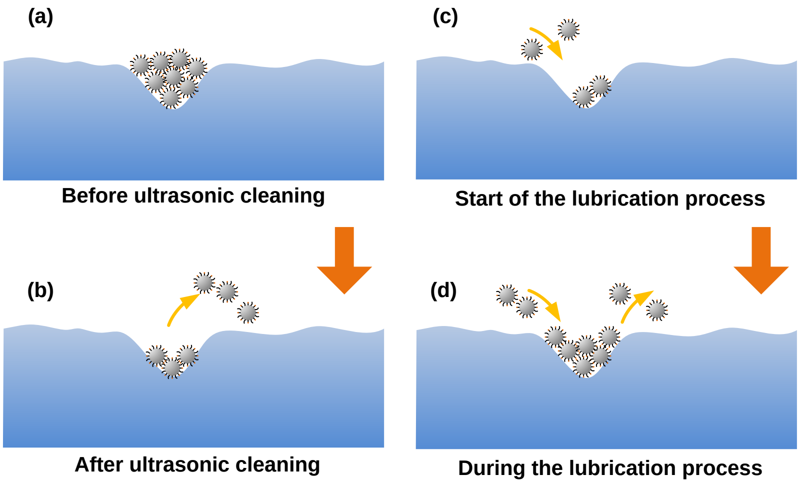

Figure S7. The nanoparticles in the groove of (a, b) before and after ultrasonic cleaning, (c) start of the lubrication process and (d) during the lubrication process.

9. Four-ball tribometer

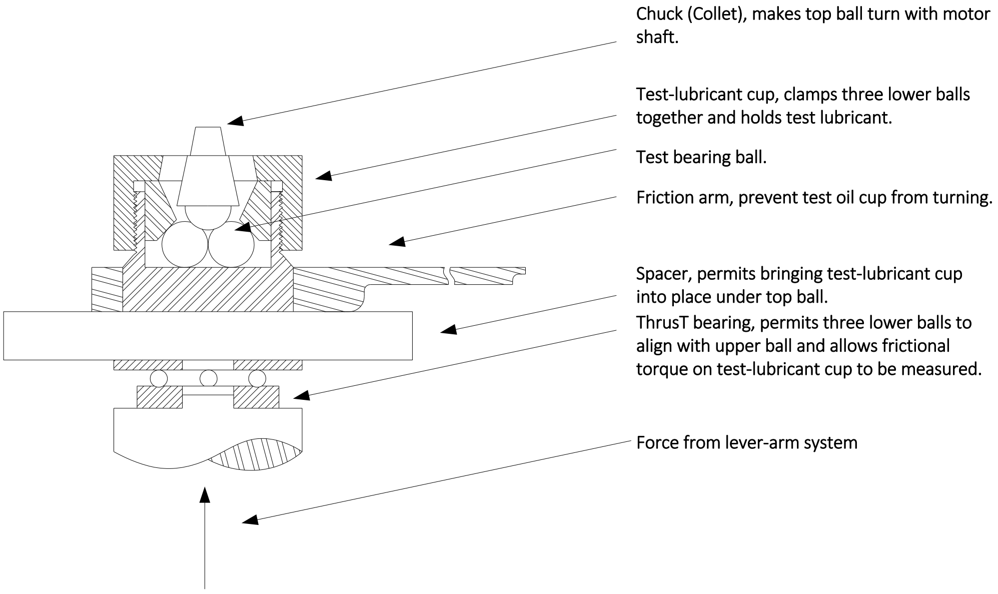

Figure S8. The section view of four-ball tribometer

The section view of four-ball tribometer was shown in Figure S8. Test procedure was list as following: Place the three test balls in the test-lubricant cup. Place the lock ring over the test balls and screw down the nut securely. Pour the lubricating fluid to be tested over the three test balls until they are covered. Press one ball into the ball chuck and mount the chuck into the chuck-holder Install the test-lubricant cup assembly on the test apparatus in contact with the fourth ball. Place the spacer between cup and thrust bearing. Place the weight tray and sufficient weights on the horizontal arm in the correct notch for a base test load. Release the lever arm and gently apply the test load to the balls, making certain the cup assembly and spacer are centered. If the optional friction-measuring device is used, connect the calibrated arm on the test-lubricant cup to the indicator spring by means of the clip and wire.
